# Three-Dimensional Morphological Characteristics of Lower Lumbar Intervertebral Foramen with Age

**DOI:** 10.1155/2018/8157061

**Published:** 2018-11-11

**Authors:** Shuaifeng Yan, Yunfan Zhang, Kai Wang, Yingchao Han, Kai Zhu, Fan He, Jun Tan

**Affiliations:** ^1^Department of Orthopaedic Surgery, Shanghai East Hospital, Tongji University School of Medicine, Shanghai, China; ^2^Department of Orthopaedic Surgery, Shanghai TCM-Integrated Hospital, Shanghai University of TCM, Shanghai 200120, China; ^3^Department of Spine Surgery, Renji Hospital, Shanghai Jiao Tong University School of Medicine, Shanghai 200127, China

## Abstract

Intervertebral foramen is the doorway of nerve root and it plays an important role of radiculopathy and surgical treatment of intervertebral foramen diseases. The purpose of the study is to obtain three-dimensional (3D) morphological characteristics of lumbar intervertebral foramen and their relationship with age. Pedicle-superior articular process (P-SAP), disc height between adjacent vertebra (DH), pedicle-inferior vertebrae (P-IV), inferior posterior vertebrae-superior articular process (IPV-SAP), and bony boundary area (BBA) were measured in entrance, middle slice, and exit of lumbar intervertebral foramen for 25 males of different age groups. Spinous process to intervertebral foramen entrance (SP-IFE) was measured for 25 males of different age groups. Overall, P-SAP and P-IV decreased and IPV-SAP increased from the entrance to the exit of intervertebral foramen for L3/4-L5S1. DH decreased at entrance slice, middle slice, and exit slice for L3/4-L5S1 with age. Significant difference with aging was found only at the middle slice of L3/4 and L4/5 for P-SAP. And the significant decrease of IPV-SAP was observed at middle slice of L3/4, entrance slice of L4/5 and L5S1, and exit slice of L5S1. SP-IFE is not consistent for all subjects. In addition, the decrease of BBA at L3/4 and L4/5 was observed earlier than at L5S1. The present study described detailed information of intervertebral foramen, which may be of benefit for better understanding of the pathology and surgical planning for intervertebral foramen diseases.

## 1. Introduction

The low back pain has been proved the first disable condition cause globally [1], it is also an important and costly medical problem that leads to decreased employee health and productivity in the workplace [2]. Radiculopathy is a common cause of low back pain [3], and it may result from compression or irritation of nerve root. Surgical treatment is a reliable and effective way to solve the compression of nerve root [4]. Compared to the traditional surgery approach, the minimally invasive surgery has a lot of advantages such as minimum post-operative pain, early return to work, shorter hospital stay, economic benefits, lesser bleeding [5–7]. Though the minimally invasive surgery is superior, due to the operation field is limited, the occurrence rate of hyperalgesia and paresthesia caused by nerve root injury is as high as 5%~15%[8]. What's more, failed back surgery syndrome (FBSS) which means low back pain doesn't get relieved or become worse after back surgery accounts for 25%~58% of the complications for those who underwent the surgery [9]. Therefore, intervertebral foramen plays an important role both in pathology of radiculopathy and surgery management. The detailed knowledge of intervertebral foramen is essential to understand potential sources of pathology and management of radiculopathy in low back pain.

It is difficult to get the direct information of three-dimensional intervertebral foramen because it has an entry and exit bounded by the medial and lateral borders of the pedicles. The bony boundary of intervertebral foramen includes adjacent vertebral pedicles superiorly and inferiorly, postero-inferior margin of the superior vertebral body, disc, and posterosuperior vertebral notch of the inferior vertebral body anteriorly [10]. Previous studies have focused on anatomical characteristics of intervertebral foramen both in vitro or vivo in spines [11–13], and many different methods have been used including silicone model of intervertebral foramen, plain radiographs, computed tomography and magnetic resonance imaging scans [14]. With development of digitization, it becomes available to explore the 3D morphological characteristics for complex anatomical structures. Some studies have attempted to explore foraminal morphological characteristics from different perspectives [10, 15, 16]. However, few studies have focus on morphological characteristic of lower back with age using 3D method.

The present work aims to provide detailed information of intervertebral foramen in healthy male individuals for different age groups at lower back. The results obtained from this study may be helpful in the better understanding of pathology and better surgery strategies for diseases related to intervertebral foramen.

## 2. Materials and Methods

### 2.1. Subject

**Figure 1:**
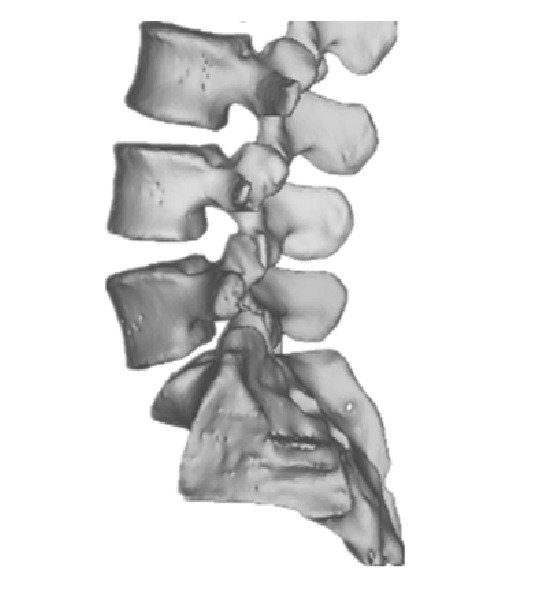
3D model of lower back (L3/4-L5S1).

The sample comprised 25 asymptomatic male volunteers (Table 1) who have signed the ethic approval which is in accordance with the ethical standards of the Medical ethics committee of Shanghai East Hospital (East Hospital Affiliated Tongji University) and with ethical standards of Medical ethics committee of Shanghai (China). All of them underwent lumbar spine CT (64-slice spiral CT, Siemens, Germany) at Shanghai East Hospital. Exclusion criteria included: current or prior back pain, anatomical abnormalities or any spinal disorders. Table 1 shows the volunteers' basic information. The CT were scanned in a supine position, and the CT images were imported into a reconstruction software (Mimics, Materialise, Inc., Leuven, Belgium).  Figure 1 shows the typical 3D model of lower back.

### 2.2. Morphological Parameters

**Figure 2:**
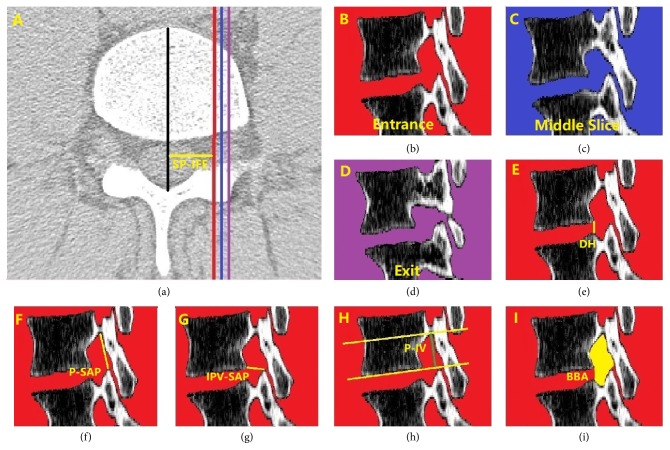
**Parameters measured in the study.** (a) The zone of closed intervertebral foramen (the red line, blue line and purple line located at the entrance, middle slice, and exit of intervertebral foramen), SP-IFE was the distance between spinous process and intervertebral foramen entrance slice; (b) the entrance slice of intervertebral foramen; (d) the exit slice of intervertebral foramen; (c) the middle sagittal slice between the entrance and exit of intervertebral foramen; The following parameters were taken at entrance slice. DH: disc height between adjacent vertebra; P-SAP: distance between pedicle and superior articular process; IPV-SAP: distance between posteroinferior margin of vertebrae and superior articular process; P-IV: distance between pedicle and inferior vertebral body; BBA: Bony boundary area of intervertebral foramina.

CT was used for anatomical measurements directly since previous studies have proved good correlation between CT measurements compared directed measurements of anatomy using cadaveric specimens in spinal anatomy [4, 9, 17]. According to Lee's study [18], the intervertebral foramen was subdivided into three zone: entrance zone, mid-zone and exit zone for common description. Similarly, three special sagittal slices of the intervertebral foramen (Figure 2(a)) were selected including the entrance (Figure 2(b)), the middle sagittal slice (Figure 2(c)) and exit (Figure 2(d)) to explore the morphology of intervertebral foramen at lower back. From spine canal to intervetebral foramen exit, we define the intervertebral foramen closed boundary that appears first as intervertebral foramen entrance, and the slice that the first closed boundary of intervertebral foramen bony boundary appears as entrance slice; we define the intervertebral foramen closed boundary that appears last as intervertebral foramen exit, and the slice that the last closed boundary of intervertebral foramen bony boundary appears last as exit slice; we could obtain the distance between entrance slice and exit slice easily in Mimics, and the midpoint of the distance can also be easily determined, then we define the sagittal slice which midpoint locates as middle slice. In fact, the location of red slice (entry slice) is not consistent for all subjects because of anatomical difference. However, we locate the red slice for each subject with a strict and unified standard as mentioned above to control research consistency. We collected parameters (DH, P-SAP, IPV-SAP, P-IV and BBA) at each sagittal slice (entrance slice, middle slice, and exit slice). And we showed how the parameters were taken at entrance slice (Figures 2(e), 2(f), 2(g), 2(h), and 2(i)). In Figure 2(a), the entrance located at the red line, the exit located at the purple line and the middle sagittal slice located at the blue line. In order to get the continuous boundary limit, a continuous line segment is used to represent the posterior margin of the intervertebral disc for bony boundary. Intervertebral foramen is a complex 3D anatomical structure, the boundary of intervertebral foramen on any sagittal slice is closed between exit sagittal slice (Figure 2(d)) and entrance sagittal slice (Figure 2(b)) of intervetebral foramen. We use Figure 2(e) as an example to show how the parameters were measured in the current study.

### 2.3. Disc Height between Adjacent Vertebra (DH), Pedicle to Articular Process Distance (P-SAP) and Posteroinferior Margin of Upper Vertebrae to Articular Process Distance (IPV-SAP)

The DH of adjacent vertebra was defined as the distance and posterior-inferior edge of upper vertebra and posterior-superior edge of lower vertebra (Figure 2(e)). The P-SAP was measured from the top of the superior articular process to the inferior aspect of the upper pedicle (Figure 2(f)). Use of the inferior cortex of the pedicle isthmus as a measurement landmark for P-SAP distance is similar to that given in the diagrams depicted in studies of the intervertebral foramen in degenerative lumbar scoliosis and thoracic intervertebral foramen osseous anatomy in adolescent idiopathic scoliosis [15]. The IPV-SAP was defined from the posteroinferior margin of upper vertebrae to superior articular process (Figure 2(g)). The P-SAP and IPV-SAP was measured as parameters to quantify the movable space of nerve root.

### 2.4. Pedicle to Inferior Vertebrae (P-IV) and Spinous Process to Intervertebral Foramen Entrance (SP-IFE)

The P-IV was defined from the inferior border of the upper pedicle to inferior border of the same vertebrae (Figure 2(h)). It is the anatomical characteristic of foramen which would not change with degeneration of intervertebral foramen. However, P-IV hasn't been described before. SP-IFE was defined as the distance between spinous process and intervertebral foramen entrance. The location of intervertebral foamen entrance is not consistent for all subjects because of anatomical difference. SP-IFE would be beneficial for readers to understand where intervertebral foramen entrance locates.

#### 2.4.1. Bony Boundary Area (BBA) of Foramen

The bony boundary area of foramen was measured using a manual cursor method encircling the margin of the posterior vertebral body, inferior border of the upper pedicle, anterior margin of the inferior articular facet, the anterior border of the superior articular facet and the superior border of the lower pedicle. Since the boundary of bony foramen is not continuous because of the disc, so in the study we draw a line from the posteroinferior margin of upper vertebrae and posterosuperior margin of lower vertebrae to get a closed bony boundary of foramen (Figure 2(i)). However, BBA described here is similar to the examples given in a lumbar study [11] and a thoracic intervertebral foramen study [8].

### 2.5. Statistical Analysis

SPSS was used for statistical analyses, and p<0.05 was considered statistically significant. ANOVA was used to compare parameters among entrance, middle slice, and exit, it was also used among different age groups for parameters and subjects (including age, height, weight, and BMI). All counting data were reported as mean ± standard error of the mean (SEM).

## 3. Results

Of the 25 male subjects enrolled in this study, a total of 150 foramen, 450 sagittal slices, and 450 closed bony boundary of intervertebral foramen were analyzed for L3/4, L4/5, and L5S1 bilaterally. The mean ± SD of foraminal parameters were shown in Tables 2 and 3. Statistically significance was found at the entrance, exit, and middle sagittal slice of foramen for P-SAP, IPV-SAP, and P-IV (Table 2).

**Figure 3:**
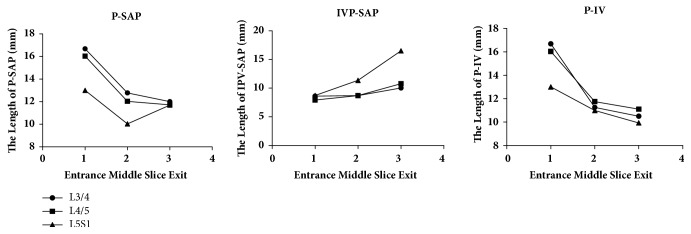
The results of P-SAP, IPV-SAP, and P-IV when grouped with different sagittal slices in males. entrance: entrance slice of intervertebral foramen; exit: exit slice of intervertebral foramen; middle slice: the sagittal slice between the entrance and exit.

**Figure 4:**
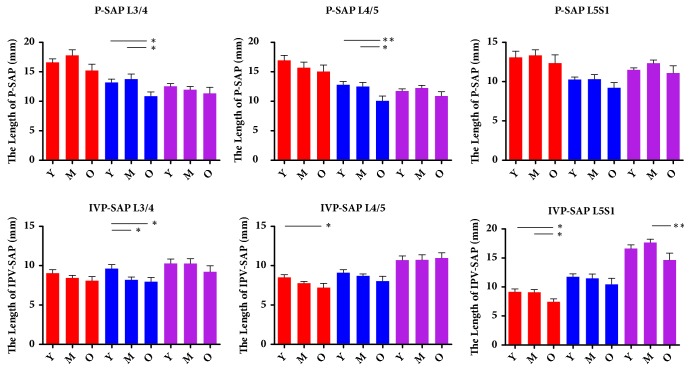
The results of P-SAP and IPV-SAP grouped with age in different sagittal slices for males. Entrance: entrance slice of intervertebral foramen; exit: exit slice of intervertebral foramen; middle slice: the sagittal slice between the entrance and exit; Y: young aged group; M: middle aged group; O: old age group. **∗** p<0.05 between two groups. **∗∗** p<0.01 between two groups.

As for P-SAP, it decreased gradually from the entrance to the exit of foramen at L3/4 and L4/5, however, at L5S1 it decreased at first then increased from the entrance to the exit of foramen (Figure 3). IPV-SAP increased gradually from the entrance to exit of foramen at L3/4, L4/5 and L5S1 (Figure 3). DH decreased at entrance slice, middle slice, and exit slice for L3/4-L5S1 with age (Table 2). P-IV decreased then kept stable from the entrance to exit at L3/4 and L4/5, but it decreased gradually from the entrance to the exit of foramen at L5S1 in which statistically significance were found among entrance, middle slice, and exit of intervertebral foramen (Figure 3). With aging, P-SAP decreased in middle sagittal slice at L3/4, L4/5. What's more, IPV-SAP decreased in entrance slice at L4/5, L5S1, in middle sagittal slice at L3/4 and in exit slice at L5S1 for males. However, no significant difference was found in other sagittal slices for P-SAP and IPV-SAP (Figure 4). P-IV hasn't been analyzed because it is the anatomical characteristics that would not change with age. SP-IFE is not consistent for all subjects, it ranged from 9.69-14.79mm at L3/4, 9.31-16.43mm at L4/5 and 12.88-20.13mm at L5S1. Significant difference of intervertebral foramen entrance was found for different lumbar levels (Table 3).

**Figure 5:**
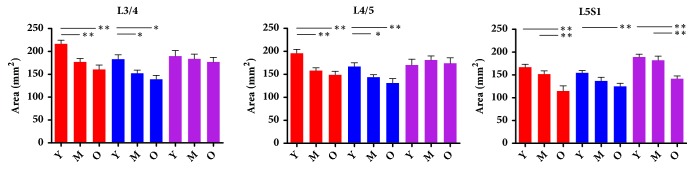
The results of BBA in different sagittal slice at L3/4-L5S1. Entrance: entrance slice of intervertebral foramen; exit: exit slice of intervertebral foramen; middle slice: the sagittal slice between the entrance and exit; Y: young aged group; M: middle aged group; O: old aged group. **∗** p<0.05 between two groups. **∗∗** p<0.01 between two groups.

The results of intervertebral foramen's BBA were shown in Figure 5. Significant difference were found for the bony boundary with age in every sagittal slice except the exit slice at L3/4 and L4/5. Overall, the BBA decreased in entrance slice, exit slice and middle sagittal slice at L3/4-L5S1 with age. Of note, the area decreased significantly from young age group to the middle age group at L3/4 and L4/5,but at L5S1 it decreased significantly from the middle age group to old age group for males (Figure 5, Table 4).

## 4. Discussion

This study aimed to use a 3D method to explore the morphological characteristics of intervertebral foramen at lower back in different age groups for males. The morphology of intervertebral foramen should centre the nerve root, so we choose the parameters that have a closed relationship with nerve root. Because the anatomical morphology of superior pedicle is fixed, the movability range of nerve root is determined by the position of the superior articular process. So we use P-SAP and IPV-SAP which had an intensive relationship with superior articular process to describe the morphological characteristics. Disc height between the adjacent verterbra would lead to 2D area and 3D volume change of intervertebral foramen. P-IV is an anatomical parameter and it is also analyzed which is determined by vertebrae itself. SP-IFE is also an anatomical parameter, which helps readers understand the location of intervertebral foamen entrance. BBA are vital because they reflect the movability range of intervertebral foramen directly in each closed intervertebral foramen boundary at each sagittal slice. BBA is the area of which boundary consists of bony anatomical structures including pedicles, vertebrae and process.

Previous studies [11, 13] always focused on one sagittal slice from which we could not get the detailed three-dimensional information of intervertebral foramen, though they have found some morphological characteristics of intervertebral foramen such as the foraminal height decreased with age and the foraminal width in both genders also decreased similarly with age [17]. Other reporters reveal the height and width of intervertebral foramen in healthy subjects and patients with degenerative lumbar scoliosis with the method of 3D [3]. Yasuhito [11] thought their research of intervertebral foramen is useful for better understanding of degenerative lumbar scoliosis. What's more, a recent report revealed the dynamic changes of dimensions of intervertebral foramen [19], and they thought human lumbar foramen dimensions show segment-dependent characteristics during the dynamic weight-lifting activity. As compared to the published studies, one of the highlights of present study is that we obtain the information from entrance to the exit of intervertebral foramen and explored the morphological parameters in different age groups.

Morphological characteristics of L3/4 and L4/5 are quite different with that of L5S1 both with and without age consideration. Without age consideration, P-SAP and P-IV decreased then kept stable from the entrance to the exit of foramen at L3/4 and L4/5, while at L5S1 P-SAP decreased then increased like “funnel.” P-IV decreased gradually like “slope” from entrance to the exit. In addition, IPV-SAP just increased gradually from entrance to the exit at different lumbar levels. With aging, P-SAP and IPV-SAP decreased with aging in some sagittal slices at different lumbar level, which indicated that superior articular process seemed more easily to get in touch the superior vertebrae including the posterior inferior part of vertebrae and lower part of pedicle with aging for males. That means the older patients' nerve root is easier to be affected than the young group and mid-aged group by superior articular process of next level's vertebra at lower back. The decrease of P-SAP and IPV-SAP might cause the compression or irritation of nerve root, which could cause low back pain and might be a pathology source of radiculopathy. The relationship between P-SAP, IPV-SAP and clinical symptoms is very meaningful in pathology of lumbar foramen stenosis, and P-SAP, IPV-SAP may be beneficial for diagnosis of lumbar foramen stenosis, which is worth to be explored in further study. DH decreased at entrance slice, middle slice, and exit slice for L3/4-L5S1 with age, which might be the cause of intervertebral foramen morphological changes with age. At L3/4 and L4/5 BBA decreased significantly from young age group to the middle age group, while no significant difference was found between the middle age group and old age group. However, it is quite different that at L5S1 the significant decrease was observed from the mid-aged to the old. The difference may result from mobile range of different lumbar levels, the moving range of L3/4 and L4/5 is larger than that of L5S1. What is more, osteophytes is a potential contributor to the decrease of BBA, which might be a source of radiculopathy.

To the best of our knowledge, the spine can perform the movement of flexion, extension, bending, and rotation. Weiye Zhong and Atsushi Fujiwara [19, 20] have demonstrated the effects of dynamic positions on the morphological characteristics. The soft tissue structures were considered important in the pathology of radiculopathy [10, 21]. The limitation of this study is that CT data included were taken in supine position and the soft tissue was not taken into consideration. So the information of dynamic positions for intervertebral foramen is not available. What's more, the spine consists of cervical vertebrae, thoracic vertebrae, and lumbar vertebrae. The present study provided detailed information of intervertebral foramen at the lower back, and the intervertebral foramen of other levers should also be taken into consideration.

In summary, the present study described foraminal morphological characteristics in different age groups for males at lower back with 3D method. Although the geometry is limited to the supine position, such information is still valuable for the better understanding of intervertebral foramen diseases, which is helpful for its pathology and surgery planning.

## Figures and Tables

**Table 1:** Subjects information of age groups.

	n	Male (25)	Height(cm)	Weight(kg)	BMI(kg/m^2^)
Young Age Group (20-40)	10	28.8±5.33	176.3±3.62	79.62±6.07	25.6±1.41
Middle Age Group (40-60)	9	47.67±3.53	175.22±3.56	77.01±8.32	25.02±1.84
Old Age Group (>60)	6	69.17±3.53	174.17±2.32	75.48±6.42	24.95±2.15

**Table 2:** Results of P-SAP, IPV-SAP, DH and P-IV grouped in different sagittal slices for males.

			Male		
			Entrance	Middle Slice	Exit
P-SAP (mm)	L3/4	Young Age Group	16.6±2.61	13.19±2.44▲	12.54±1.96■●
		Middle Age Group	17.85±4.04	13.66±3.64▲	11.82±2.27●
		Old Age Group	15.21±3.57	10.87±2.37▲◆★	11.35±3.43●
	L4/5	Young Age Group	16.94±3.63	12.79±2.45▲	11.75±1.60●
		Middle Age Group	15.67±3.95	12.51±2.79▲◆★	12.23±1.93●
		Old Age Group	15.07±3.54	10.07±2.73▲	10.89±2.47●
	L5S1	Young Age Group	13.10±3.37	10.28±1.34▲	11.49±1.16■
		Middle Age Group	13.33±2.98	10.31±2.42▲	12.34±1.66■
		Old Age Group	12.36±3.46	9.22±2.15▲	11.09±3.04

IPV-SAP (mm)	L3/4	Young Age Group	9.05±1.95	9.63±2.22	10.28±2.45
		Middle Age Group	8.44±1.33	8.19±1.46▲◆	10.27±2.52■
		Old Age Group	8.08±1.75	7.9±1.75★	9.22±2.54
	L4/5	Young Age Group	8.50±1.50	9.11±1.68▲	10.71±2.25■●
		Middle Age Group	7.77±0.92	8.69±1.01▲	10.73±2.66■●
		Old Age Group	7.22±1.75★	8.03±2.04	10.97±2.20■●
	L5S1	Young Age Group	9.14±2.11	11.63±2.12▲	16.63±2.76■●
		Middle Age Group	9.11±1.93	11.63±3.20▲	17.78±2.29■●
		Old Age Group	7.46±1.66◆★	10.46±3.46▲	14.66±3.92■●◆

DH (mm)	L3/4	Young Age Group	5.17±1.08	5.47±0.93	5.92±1.14
		Middle Age Group	5.04±0.66	5.02±0.74	5.65±0.91
		Old Age Group	2.99±1.27◆★	3.36±1.06◆★	4.13±1.37◆★
	L4/5	Young Age Group	4.63±0.95	4.81±1.00	5.60±1.17
		Middle Age Group	5.23±0.97	5.42±0.92	6.11±1.07
		Old Age Group	3.20±1.33◆★	3.71±1.29◆★	4.64±1.49★
	L5S1	Young Age Group	3.64±0.59	4.11±0.77	5.66±1.19
		Middle Age Group	3.57±1.43	3.69±1.32	4.76±1.29☆
		Old Age Group	1.12±0.73◆★	1.08±0.76◆★	1.39±1.13◆★

P-IV (mm)	L3/4		14.88±2.52	11.25±2.23▲	10.50±2.23●
	L4/5		15.20±2.74	11.75±1.97▲	11.10±1.39●
	L5S1		14.38±3.12	10.99±1.97▲	9.93±1.54■●

▲p<0.05 Compared with entrance ■p<0.05 Compared with middle slice ●p<0.05 Compared with entrance

☆p<0.05 Compared with the young group ◆p<0.05 Compared with the young group ★p<0.05 Compared with mid-aged group

**Table 3:** Results of SP-IFE grouped in different lumbar level for males.

		Male	
		Entrance	Range
SP-IFE(mm)	L3/4	12.48±1.10	9.69-14.79
	L4/5	13.52±1.49☆	9.31-16.43
	L5S1	12.48±1.10◆★	12.88-20.13

☆p<0.05 Compared with the young group ◆p<0.05 Compared with the young group ★p<0.05 Compared with mid-aged group

**Table 4:** The bony boundary area of different age groups at different sagittal slices.

		Bong Boundary Area (mm^2^)		
		Young Age Group	Middle Age Group	Old Age Group
L3/4	Entrance	216.60±35.13	176.91±31.38☆	160.52±32.52★
	Middle Slice	183.02±43.11	150.09±29.32☆	139.16±28.01★
	Exit	189.66±53.69	183.80±42.49	158.61±41.52

L4/5	Entrance	195.72±37.32	158.10±24.84☆	148.94±24.98★
	Middle Slice	166.95±35.40	143.64±22.20☆	131.23±31.58★
	Exit	170.22±54.58	181.01±36.52	174.05±39.18

L5S1	Entrance	166.90±28.17	151.94±29.26	114.69±37.87★◆
	Middle Slice	154.57±22.53	136.68±33.17	125.37±22.80★
	Exit	189.17±27.92	182.19±36.97	140.72±20.95★◆

☆p<0.05 compared to the young age group ◆p<0.05 compared to the young age group ★p<0.05 compared to middle age group

## Data Availability

All relevant data are available in supplementary materials (available here).

## Supplementary Materials

Supplementary Materials Raw data of the study.
