# Supplementary material for: Three-Dimensional Morphological Characteristics of Lower Lumbar Intervertebral Foramen with Age
Source: Biomed Res Int. 2018 Nov 11;2018:8157061. doi: 10.1155/2018/8157061 (PMC6252236; doi:10.1155/2018/8157061)
Supplement: Supplementary Materials — Raw data of the study. [file 8157061.f1.pdf]

This is raw statistical data involved in the study.

R:Right side L:Left side

	Parameter	Level	Slice	1R	2R	3R	4R	5R	6R	7R	8R	9R	10R	1L	2L	3L	4L	5L	6L	7L	8L	9L	10L
			ntranc	15.67	15.06	22.4	15.87	20.65	18.28	13.24	15.18	17.72	15.59	13.9	14.66	17.42	12.7	20.12	18.28	13.57	18.37	18.88	14.45
		L3/4	id-slic	11.67	11.57	19.02	11.31	16.69	14.57	8.98	11.54	11.73	13.02	11.32	14.25	14.5	9.21	15.56	14.57	12.41	13.38	16.21	12.3
			exit	10.08	11.73	15.11	9.23	14.51	13.12	12.44	15.02	11.36	12.73	9.37	13.12	14.38	8.82	15.58	13.12	11.69	13.72	13.58	12.17
			ntranc	20.45	12.67	22.25	17.66	19.39	20.6	14.36	10.99	9.06	17.64	16.93	20.77	15.53	16.21	22.46	20.6	17.29	16.1	14.26	13.66
	P-SAP	L4/5	id-slic	13.1	10.74	18.24	11.89	14.25	15.4	10.94	10.37	8.22	13.17	12.95	15.62	14.4	9	15.23	15.4	12.21	12.43	10.09	12.12
			exit	10.64	10.37	15.31	9.23	12.6	12.77	12.18	10.72	10.93	13.37	11.4	12.32	13.93	8.28	13.21	12.77	11.33	11.04	10.57	11.93
			ntranc	9.12	12.85	19.65	12.31	9.7	14.09	10.63	10.38	9.97	13.47	10.31	21.89	10.34	11.01	15.16	14.09	16.35	15.05	15.52	10.06
		L5S1	id-slic	7.97	11.6	10.99	9.06	10.87	10.27	9.36	10.05	11.28	10.66	8.67	12.46	7.98	8.7	9.47	10.27	11.81	10.43	12.66	10.94
			exit	12.07	11.11	11.6	11.41	12.67	10.79	9.48	12	14.41	9.94	12.04	12.71	10.24	10.53	11.97	10.79	10.19	11.48	13.07	11.31
			ntranc	6.38	9.08	8.86	8.28	12.43	9.61	6.13	11.97	7.77	9.16	5.7	9.69	8.76	8.71	12.74	9.61	6.38	11.2	9.24	9.27
		L3/4	id-slic	4.92	9.56	9.37	8.12	11.77	12.21	6.66	12.19	7.09	7.4	8.64	12.21	11.76	8.17	11.36	12.21	7	11.86	9.63	10.38
			exit	5.86	12.85	9.99	10.09	11.11	11.8	7.4	15.03	9.32	6.44	8.98	10.8	12.44	8.24	11.38	11.8	6.82	14.41	11.05	9.85
			ntranc	6.91	9.77	8.6	8.45	9.12	9.16	6.2	11.05	9.6	7.73	5.97	9.15	7.96	7.3	11.06	9.16	5.89	9.96	7.62	9.43
	IPV-SAP	L4/5	id-slic	6.57	11.26	11.04	8.74	9.14	9.07	7.45	12.43	9.91	8.81	6.13	9.57	10.27	8.47	8.66	9.07	5.68	11.07	9.17	9.66
			exit	6.8	11.71	13.03	9.73	8.84	13.92	8.46	15.03	12	9.28	7.68	9.86	11.91	9.53	10.58	13.92	7.22	11.47	12.03	11.21
			ntranc	7.79	11.49	8.29	7.08	8.53	9.5	7.78	13.42	12.35	6.59	8.92	9.07	8.82	5.9	6.36	9.5	7.07	12.16	13.06	9.38
		L5S1	id-slic	13.73	14.35	10	10.17	11.92	11.59	11.84	16.04	15.55	8.15	11.42	10.37	11.75	8.83	9.91	11.59	8.25	13.87	13.38	12.3
			exit	20.24	15.91	15.48	15.48	16.51	16.88	13.93	21.11	20.51	9.16	19.35	17	18.91	16.15	17.55	16.88	13.18	16.18	18.47	13.52
			ntranc	15.19	13.42	18.32	14.55	11.61	14.23	11.78	13.47	17.91	14.46	10.16	14.23	13.14	11.85	10.87	14.23	12.71	17.73	19.23	13.02
		L3/4	id-slic	10.22	9.41	15.76	10.35	9.38	11.16	7.92	11.47	12.35	11.2	7.97	11.16	10.41	8.7	8.63	11.16	11.31	12.66	15.93	9.89
			exit	8.89	9.79	13.05	9.45	8.64	11.18	11.93	13.6	10.46	10.24	7.09	11.02	9.75	8.55	8.94	11.18	10.54	11.74	11.62	9.16
			ntranc	18.38	11.04	19.34	18.72	14.88	16.73	14.26	11.46	11.02	16.15	14.19	18.45	13.1	13.84	14.03	16.73	17.25	17.29	16.35	10.49
	P-IV	L4/5	id-slic	11	10.54	15	11.18	10.63	13.44	10.11	10.16	9.67	12.3	10.57	14.17	10.34	10.9	11.52	13.44	13.86	14.26	12.16	8.79
Young Group			exit	9.12	9.25	11.83	10.27	8.57	11.31	11.98	11.74	9.74	11.82	9.55	12.05	9.62	8.61	9.26	11.31	11.74	11.38	11.64	9.18
			ntranc	11.91	12.76	16.8	13.17	7.87	13.78	12.01	12.94	11.62	14.23	13.21	20.32	13.25	13.19	16.82	13.78	15.61	19.21	18.19	10.26
		L5S1	id-slic	8.1	9.41	12.8	10.29	9.84	11.67	9.41	10.45	10.54	11.34	9.15	13.61	11.36	9.06	12.19	11.67	13.57	13.35	13.57	10.26
			exit	8.31	9.85	12.36	8.84	7.01	10.42	9.92	11.46	9.18	9.93	7.77	10.09	12.2	8.49	9.57	10.42	10.74	10.47	11.05	9.74
			ntranc	5.79	5.37	5.94	4.23	6.76	4.91	3.47	6.63	4.92	3.98	6.9	5.06	5.34	3.88	6.61	4.56	4.39	6.41	4.11	4.21
		L3/4	id-slic	5.51	6.16	5.57	4.38	6.45	5.12	4.18	5.75	5.97	4.65	6.27	5.33	5.69	4.23	7.24	6.61	4.22	6.63	4.97	4.43
			exit	6.9	6.5	5.93	4.8	7.56	5.57	4.56	6.18	6.98	5.98	6.48	5.55	5.95	4.02	8.14	6.67	3.44	6.16	5.35	5.76
			ntranc	5.45	4.37	4.92	4.38	4.56	4.39	3.63	4.71	3.49	3.98	6.04	5.93	5.55	4.02	6.61	4.71	3.8	3.91	2.9	5.32
	DH	L4/5	id-slic	6.79	5.01	5.24	4.38	5.21	5.43	3.75	3.54	3.83	5.32	6.05	5.09	5.08	4.02	6.62	5.21	3.57	4.43	3.18	4.48
			exit	7.26	5.76	5.56	5.41	7.12	5.73	4.45	3.49	4.76	4.91	7.42	5.61	6.19	4.98	7.71	6.86	4.07	5.34	4.73	4.67
			ntranc	3.57	3.41	3.89	3.01	4.6	2.67	2.89	3.67	3.53	3.37	3.63	3.79	4.28	2.91	4.92	4.39	3.08	3.99	3.63	3.55
		L5S1	id-slic	3.83	3.75	4.63	3.76	4.76	4.21	2.38	3.58	3.24	4.56	4.23	4.28	4.49	4.97	5.21	5.71	3.44	3.27	3.65	4.3
			exit	6.88	6.39	7.12	6.26	5.97	5.18	5.03	3.27	3.78	5.25	5.98	6.14	7.31	6.05	6.47	7.31	3.69	5.71	4.85	4.51
			ntranc	205.2	211	261.8	179.9	240.5	262.7	163	235.2	205.2	166.6	228.4	211.6	247.3	184.3	257.7	262.72	157.59	249.58	228.43	173.06
		L3/4	id-slic	159.4	195.4	222	132.1	195.1	232.2	106.1	115.2	159.4	139.4	211.2	211.2	251	152	221.9	232.18	137.27	225.14	211.22	150.88
			exit	216	234.9	219.5	143.1	203.3	223	199.3	5.95	216	146.5	196.7	196.7	251.8	154.7	236.8	222.95	133.2	231.74	196.65	164.52
			ntranc	198.88	203.24	216.01	198.88	175.05	281.7	154.1	158.7	150.41	194.81	163.6	236.47	204.94	163.6	227.85	281.69	182.44	192.34	158.99	170.74
	BBA	L4/5	id-slic	147.01	213.81	208.85	147.01	155.15	233.3	119.9	155	142.31	158.32	124.63	203.74	203.13	124.63	161.62	233.34	138.25	177.67	135.34	155.97
			exit	147.78	216.97	219.2	147.78	173.57	271.3	174.2	12.39	149.2	147.36	130.87	175.89	216.91	130.87	199.95	271.3	144.17	171.78	145.47	157.43
			ntranc	187.7	189	204	121.1	139.2	187.7	134.7	142.2	164.8	159.3	187.7	216.7	150	104.9	172.3	187.69	177	174.39	190.03	147.62
		L5S1	id-slic	168	176.8	158.8	112.9	152.1	168	124	151.2	179.9	133.8	168	151.1	158.9	97.64	156.9	167.96	152.05	148.61	194.3	170.65
			exit	205.7	259.5	211.8	149.4	169.3	205.7	170.3	183	199.1	142.1	205.7	215.5	213.8	166.9	177.7	205.68	162.76	152.02	206.33	181.34
		L3/4		12.02	16.67	20.13	15	18.18	18.62	14.97	15.03	16.1	14.92	13.57	16.59	19.08	14.48	17.88	18.78	15.68	15.74	15.12	18.01
	SP-IFE	L4/5		9.31	14.94	16.43	12.55	15.71	15.01	13.54	13.28	14.25	12.53	12.04	14.39	16.43	12.99	15.97	14.2	12.85	13.84	14.73	15.45
		L5S1		9.69	13.21	13.29	11.03	12.08	12.53	12.48	12.31	11.41	11.23	11.63	13.25	14.83	12.53	13.94	13.02	11.34	12.57	12.39	12.6

Parameter	Level	Slice	1R	2R	3R	4R	5R	6R	7R	8R	9R	1L	2L	3L	4L	5L	6L	7L	8L	9L
		ntranc	13.44	18	11.58	19.64	26.67	11.75	19.5	19.86	18.52	19.23	10.62	19.71	23.32	16.98	18.45	19.63	16.55	16.52
	L3/4	id-slic	10.32	13.09	10.11	17.18	20.95	11.03	12.8	14.05	10.5	12.71	7.92	18.03	21.01	12.74	12.63	15.76	11.39	15.32
		exit	9.13	13.21	10.88	12.27	16.11	10.63	13.62	11.47	8.59	11.98	8.24	12.71	16.97	10.97	10.6	12.62	11.02	14.12
		ntranc	13.62	19.81	10.91	23.04	16.33	12.59	13.9	14.6	16.11	14.12	15.64	8.82	15.82	25.15	10.96	17.46	16.2	17.26
P-SAP	L4/5	id-slic	10.52	13.52	9.2	15.8	14.63	11.52	10.53	12.62	11.89	12.25	11.19	8.46	12.25	21.64	11.43	13.02	12.77	11.92
		exit	10.94	12.12	12.4	11.8	14.28	11.34	11.12	12.38	13.9	11.75	11.85	7.79	12.25	17.31	11.03	12.35	10.81	14.8
		ntranc	15.07	15.74	12.55	12.7	15.74	11.45	9.61	12.9	11.8	14.99	10.16	7.26	16.49	21.13	12.93	13.44	11.58	14.33
	L5S1	id-slic	12.48	8.57	6.88	10.37	16.38	10.25	9.92	8.85	13.03	10.83	9.69	9.38	9.68	14.75	6.76	8.74	8.6	10.38
		exit	11.92	12.9	12.22	13.44	15.23	12.78	11.79	10.68	12.4	11.49	14.27	13.61	11.64	15.35	9.39	11.55	12.51	9
		ntranc	6.35	8.4	8.34	7.02	11.04	6	10.27	8.35	7.95	7.82	9.78	7.4	8.79	9.82	7.64	10.03	9.07	7.92
	L3/4	id-slic	5.12	7.98	9.33	7.66	10	6.29	9.63	7.58	7.5	6.94	8.96	9.45	8.81	8.96	6.12	11.06	7.97	8.09
		exit	7.37	10.49	13.49	9.13	9.94	6.2	13.4	9.17	13.53	8.17	11.2	9.93	9.92	10.22	7.23	16.55	9.46	9.39
		ntranc	8.14	7.66	6.42	8.3	7.67	7.96	7.96	6.7	7.41	9.2	9.08	7.09	8.1	7.09	5.65	8.75	7.88	8.87
IPV-SAP	L4/5	id-slic	7.33	8.55	9.56	7.01	8.21	9.78	9.42	8.23	10.5	9.51	9.56	8.11	9.28	7.87	7.81	9.22	7	9.53
		exit	9.16	10.45	13.32	9.7	8.21	12.4	14.02	7.88	16.22	10.53	11.3	8.34	11.96	8.3	7.63	11.36	7	15.39
		ntranc	9.71	8.31	7.49	12.39	10.16	7.17	10.44	7.41	12.57	9.6	7.9	7.13	7.87	10.88	5.95	11.09	9.23	8.41
	L5S1	id-slic	10.69	10.12	12.5	14.81	11.72	13.36	13.87	10.44	15.84	10.9	11.19	13.63	1.46	11.83	9.55	14.42	11.25	9.28

			exit	14.07	19.44	20.55	19.87	13.52	17.68	16.26	16.7	19.93	18.18	22.12	19.57	16.57	16.86	16.84	18.05	18.39	13.34
			ntranc	13.65	13.56	14.8	19.69	19.28	9.55	14.11	18.18	15.53	16.03	14.83	13.57	15.66	19.6	15.75	14.58	16.44	13.38
		L3/4	id-slic	9.81	9.67	9.59	15.48	14.36	9.69	10.19	11.37	8.3	10.12	10.69	11.24	11.71	17.76	10.84	9.77	13.29	9.11
			exit	8.43	9.18	9.04	12.61	12.26	9.69	10.89	9.27	13.38	9.22	9.54	10.28	9.88	14.37	10.61	11.28	11.02	9.77
			ntranc	12.09	17.49	14.51	18.72	12.74	10.97	13.29	13.13	14.81	13.03	9.81	11.8	13.64	20.62	16.14	16.23	16.44	15.64
	P-IV	L4/5	id-slic	10.04	12.6	9.6	15.42	10.87	10.22	10.03	11.91	8.55	9.63	9.36	11.69	11.86	18.39	11.53	11.98	12.59	10.79
Mid-aged Group			exit	10.99	11.15	13.22	12.3	10.36	10.2	10.37	10.53	10.41	10.41	9.47	10.21	10.19	14.27	10.85	11.57	10.86	10.81
			ntranc	15.22	16.42	15.44	12.78	14.08	12.43	10.7	13.87	9.76	14.75	12.33	11.21	20.18	19.52	15.97	15.22	13.28	12.29
		L5S1	id-slic	10.21	10.17	10.06	9.85	12.66	9.72	7.2	8.41	8.32	9.82	9.37	11.22	13.45	16.09	10.21	9.47	10.91	10.02
			exit	10.29	9.78	11.23	9.05	11.83	8.86	6.63	9.46	9.4	9	7.3	9.54	10.1	11	7.51	8.45	8.44	10.06
			ntranc	3.96	4.67	5.15	5.44	5.64	4.8	5.01	5.67	4.79	5.51	5.32	4.68	5.9	5.83	3.89	4.52	5.93	4.07
		L3/4	id-slic	5.53	4.37	5.05	5.89	6.34	3.77	4.2	5.29	5.13	5.37	4.67	5.61	5.93	5.29	4.23	4.05	5.42	4.28
			exit	5.57	5.01	6.55	6.25	7.13	4.04	4.68	6.07	4.95	7.27	5.16	6.42	6.45	5.78	4.53	4.87	5.32	5.72
			ntranc	4.88	4.87	4.04	5.37	6.49	5.04	3.73	5.73	4.72	6.37	5.49	3.76	6.3	6.97	6.11	4.11	5.29	4.82
	DH	L4/5	id-slic	5.51	5.18	4.85	6.59	6.28	4.93	3.98	5.81	5.29	6.22	5.26	4.64	5.81	6.96	6.6	3.45	5.08	5.1
			exit	6.55	5.02	5.31	7.12	6.46	5.83	5.6	6.33	5.44	7.13	5.93	4.96	6.95	8.33	7.54	3.99	5.17	6.38
			ntranc	4.51	3.69	4.46	3.73	5.6	3.14	0.5	2.76	3.28	4.85	3.1	4.01	3.98	6.56	2.73	1.49	2.07	3.79
		L5S1	id-slic	4.73	3.08	4.12	4.33	5.53	2.9	1.03	3.82	3.46	4.47	3.51	3.67	4.03	6.44	2.98	1.05	3.06	4.16
			exit	5	5.4	4.29	6.15	5.27	4.17	1.97	4.32	4.2	5.17	6.6	4.12	6.19	6.88	4.01	2.35	5.03	4.6
			ntranc	145	175.2	130.9	189.7	241.4	130.2	165.2	190.4	164.5	185.8	194	142	205.3	236.3	154.9	154.17	202.84	176.52
		L3/4	id-slic	123.1	139.4	117.3	181.5	216.4	124.7	134.2	149.3	155.4	124.3	155.3	123.3	195.2	197.7	131.9	145.38	183.9	139.54
			exit	129.4	168.4	126.8	182.7	206.8	137	200	180.2	304.2	173.9	166.2	132.4	196.9	198.1	180.2	227.63	161.91	236
			ntranc	142.43	178.82	108.28	187.33	147	151	143.1	152.3	170.46	151.25	160.13	112.19	173.55	207.54	140.37	154.55	174.96	190.56
	BBA	L4/5	id-slic	119.74	145.42	99.69	159.83	155.66	168	110.6	144.4	157.5	136.24	141.17	114.68	171.52	184.1	156.78	120.98	151.64	147.62
			exit	145.37	151.33	217.17	174.34	157.64	215.2	186.2	176.9	276.3	192.08	161.57	120.38	190.22	165.37	201.06	132.31	167	227.77
			ntranc	175.3	161.5	120.1	145.5	197.1	134.2	117.1	147.7	168.1	172.5	147	105.2	171.6	220.1	111.9	136.28	138.22	165.53
		L5S1	id-slic	133.8	143.9	94.03	142.1	195.9	134.9	107.9	98.55	191.8	146.8	143.7	112.1	154	206.9	104	96.61	116.79	136.49
			exit	171.1	191.8	205.3	188.9	206.3	163.6	112.8	139	238.5	209.4	238.1	173.4	170	250.4	141	147.11	145.76	187.26
		L3/4		16.63	13.04	13.41	17.4	16.73	15.25	12.98	13.79	15.96	15.52	14.73	14.39	14.52	16.87	14.39	13.38	14.18	14.61
	SP-IFE	L4/5		14.2	11.91	11.01	14.35	13.69	14.82	12.58	13.76	12.14	15.26	12.14	12.14	15.13	13.12	14.74	13.02	13.79	12.37
		L5S1		11.88	11.01	10.34	12.9	13.12	13.71	12.19	12.22	11.92	10.97	10.79	11.72	13.35	12.36	12.99	11.98	11.8	13.26
	Parameter	Level	Slice	1R	2R	3R	4R	5R	6R	1L	2L	3L	4L	5L	6L						
			ntranc	12.78	13.16	17.88	11.11	14.5	22.09	15.34	17.26	9.74	11.49	18.74	18.46						
		L3/4	id-slic	10.32	11.57	14.42	6.43	9.92	10.62	10.11	13.16	10.18	7.18	14.34	12.15						
			exit	9.89	9.57	10.6	6.88	9.33	10.29	8.3	14.71	9.3	11.66	18.74	16.9						
			ntranc	15.23	15.5	20.4	14.18	8.25	16.07	19.31	18.36	8.73	14.66	16.53	13.57						
	P-SAP	L4/5	id-slic	9.12	11.45	13.73	5.71	7.17	11.7	12.98	12.67	6.57	6.88	12.38	10.48						
			exit	16.03	11.44	8.82	11.19	12.37	12.16	10.83	13.9	8	6.77	9.13	10						
			ntranc	10.99	11.2	7.49	7.23	10.17	10.8	19.11	17.49	12.59	12.15	13.58	15.49						
		L5S1	id-slic	9.91	9.53	7.11	9.86	13.15	8.01	10.66	12.69	6.32	7.98	9.21	6.21						
			exit	12.41	9.04	11.2	13.73	17.61	9.65	7.92	12.38	5.19	9.46	11.37	13.11						
			ntranc	6.12	5.86	6.69	7.86	9.1	9.64	5.62	9.17	7.45	8.17	9.83	11.5						
		L3/4	id-slic	4.56	8.1	6.69	6.42	7.19	9.48	6.73	8.17	9.33	9.16	8.11	11.63						
			exit	5.94	9.05	6.6	7.12	9.03	11.34	7.32	9.82	7.27	12.39	9.83	14.91						
			ntranc	5.25	5.6	8.43	7.86	9.56	9.12	6.92	5.54	5.94	4.88	7.26	10.26						
	IPV-SAP	L4/5	id-slic	4.27	8.68	7.9	5.22	10.5	10.06	6.68	11.11	7.66	9.29	6.12	8.91						
			exit	9.47	12.74	8.69	10.46	14.65	12.52	8.55	14.52	9.54	8.55	9.34	12.56						
			ntranc	8.42	4.71	7.78	7.11	9.36	5.94	6.2	6.91	8.83	7.02	11.07	6.18						
		L5S1	id-slic	6.56	6.79	13.21	8.46	10.83	8.88	12.41	7.47	9.14	12.97	19.36	9.49						
			exit	8.18	11.6	12.59	16.63	17.07	19.43	12.37	9.46	14	16.92	22.25	15.44						
			ntranc	12.69	13.94	19.77	15.53	17.14	15.64	16.19	15.67	11.51	14.91	18.3	12.54						
		L3/4	id-slic	10.03	11.69	15.91	10.9	12.57	10.54	11.09	11.32	12.48	9.75	16.19	10.18						
			exit	11.01	9.8	11.72	9.11	1.85	10.18	9.22	10.02	12.61	10.51	18.3	12.91						
			ntranc	13.74	15.07	21.1	15.53	13.93	14.99	17.46	17.9	13.38	18.71	19.74	13.65						
	P-IV	L4/5	id-slic	9.73	11.32	14.24	11.08	13.89	11.11	12.79	10.93	11.15	12.82	16.32	11.16						
Young Group			exit	13.73	11.7	12.87	12.96	13.68	10.31	10.93	12.82	13.21	10.07	13.24	11.54						
			ntranc	10.5	17.04	13.03	9.99	12.86	11.63	14.04	20.9	16.95	17.67	22.36	15.64						
		L5S1	id-slic	10.25	14.07	9.33	10.7	13.46	10.92	10.17	15.13	11.19	9.61	15.78	9.96						
			exit	9.49	11.49	8.85	10.12	13.08	10.75	9.23	13.26	9.82	10.66	14.13	10.06						
			ntranc	4.01	3.58	2.69	3.25	3.35	2.38	3.38	4.05	5.22	0.5	1.52	1.92						
		L3/4	id-slic	3.66	4.13	3.11	3	2.46	3.16	4.99	4.02	5.07	1.54	2.47	2.71						
			exit	5.78	4.71	3.39	4.18	3.2	3.94	6.1	5.23	5.32	1.34	3.23	3.16						
			ntranc	3.87	3.49	4.91	1.27	1.22	4.54	2.72	3.63	4.14	1.02	3.8	3.73						
	DH	L4/5	id-slic	3.31	2.81	5.47	2.69	1.31	5.66	4.25	4.59	3.65	2.32	4.11	4.32						
			exit	4.68	5.57	6.47	3.91	1.7	6.23	5.13	4.36	4.65	2.12	4.87	5.98						
			ntranc	0.65	2.21	0.59	0.98	0.69	0.95	0.42	2.66	1.81	1.35	0.44	0.72						
		L5S1	id-slic	0.35	1.71	0.38	1.02	0.53	0.78	0.54	2.25	2.63	1.45	0.58	0.75						
			exit	0.51	2.6	0.32	1.55	0.32	0.71	0.68	3.05	3.59	1.8	0.73	0.84						
			ntranc	132.7	137	223.5	127	165.2	200.2	144.2	167.9	164.7	111.9	203.3	148.8						

	L3/4	id-slic	125.5	135.2	179	88.97	138.5	141	128.5	145.3	146.7	93.81	188.7	158.7
		exit	113.2	133.6	157.4	94.68	154.6	215.1	112.1	206.6	137.3	162.8	186.9	229
		ntranc	125.09	132.28	200.65	136.59	103.17	183.7	158.7	136.1	161.07	141.91	154.82	153.19
BBA	L4/5	id-slic	115.61	115.34	190.2	75.04	94.58	153.8	141	138.8	158.91	93.98	154.43	143.1
		exit	167.32	207.1	161.93	170.98	142.38	242.2	147.9	234.5	169.83	104.09	137.08	203.22
		ntranc	111	121.7	105.5	78.33	13.72	115.9	103.9	146.1	161.3	127.9	154.3	137.8
	L5S1	id-slic	130.3	120.3	92.13	91.89	138.6	101.1	103.7	142.2	160.6	124.7	157	136.2
		exit	189.4	138.8	110.7	121.9	130.7	122.9	145.3	144.3	133.1	144.2	166.8	152.4
	L3/4		13.99	14.42	16.23	14.33	17.79	16.34	12.88	12.88	16.12	13.48	17.28	16.69
SP-IFE	L4/5		12.59	13.2	14.69	11.96	13.48	12.26	11.41	12.58	15.38	11.29	13.06	13.59
	L5S1		12.81	14.73	12.84	13.08	13.09	11.26	12.2	13.59	14.79	13.71	13.69	12.4

		Young Group									
	Age	35	31	22	35	36	29	24	20	29	27
	Height	175	173	177	174	179	176	173	177	174	185
	Weight	82.5	70.1	82.2	76.8	78.5	83	70.4	79.6	83.1	90
	BMI	26.94	23.42	26.24	25.37	24.5	26.8	23.52	25.41	27.45	26.3
		Mid-aged Group									
	Age	47	50	40	49	50	49	51	43	50	
Basic informati	Height	172	174	176	178	170	173	175	177	182	
	Weight	66	72.4	80.3	74.5	68.6	76.7	77.8	82.8	94	
	BMI	22.31	23.91	25.92	23.51	23.73	25.63	25.4	26.43	28.38	
		Old Group									
	Age	74	65	64	71	70	71				
	Height	177	171	176	174	175	172				
	Weight	80.5	69.9	75.4	68.1	73.9	85.1				
	BMI	25.7	23.9	24.7	22.49	24.13	28.77				
